# Ocular Blood Flow and Normal Tension Glaucoma

**DOI:** 10.1155/2015/308505

**Published:** 2015-10-19

**Authors:** Ning Fan, Pei Wang, Li Tang, Xuyang Liu

**Affiliations:** ^1^Shenzhen Key Laboratory of Ophthalmology, Shenzhen Eye Hospital, Jinan University, Shenzhen 518040, China; ^2^Department of Ophthalmology, West China Hospital, Sichuan University, Chengdu 610041, China

## Abstract

Normal tension glaucoma (NTG) is known as a multifactorial optic neuropathy characterized by progressive retinal ganglion cell death and glaucomatous visual field loss, even though the intraocular pressure (IOP) does not exceed the normal range. The pathophysiology of NTG remains largely undetermined. It is hypothesized that the abnormal ocular blood flow is involved in the pathogenesis of this disease. A number of evidences suggested that the vascular factors played a significant role in the development of NTG. In recent years, the new imaging techniques, fluorescein angiography, color Doppler imaging (CDI), magnetic resonance imaging (MRI), and laser speckle flowgraphy (LSFG), have been used to evaluate the ocular blood flow and blood vessels, and the impaired vascular autoregulation was found in patients with NTG. Previous studies showed that NTG was associated with a variety of systemic diseases, including migraine, Alzheimer's disease, primary vascular dysregulation, and Flammer syndrome. The vascular factors were involved in these diseases. The mechanisms underlying the abnormal ocular blood flow in NTG are still not clear, but the risk factors for glaucomatous optic neuropathy likely included oxidative stress, vasospasm, and endothelial dysfunction.

## 1. Introduction

Normal tension glaucoma (NTG) is known as a multifactorial optic neuropathy characterized by progressive retinal ganglion cell (RGC) death and glaucomatous visual field loss. Despite the fact that the IOP in NTG patients is within the normal range, the glaucomatous optic neuropathy may keep getting worse progressively and irreversibly. The disturbed ocular blood flow is a significant factor in pathogenesis of NTG. The vascular failure including vasospasms, small vessel disease, or autoregulatory dysfunction will lead to perfusion deficits of the optic nerve head, retina, and choroid and furthermore develop to the glaucomatous optic neuropathy [1]. Previous studies showed that the impaired vascular autoregulation was more pronounced in NTG than in high tension glaucoma (HTG), especially in NTG patients with progressive optic neuropathy than those with relatively stable status [2, 3]. Therefore, the ocular blood flow deficit may play a significant role in the glaucomatous optic neuropathy in NTG.

## 2. The Anatomy of Blood Supply of Optic Nerve

The blood supply of the eye is mainly from the ophthalmic artery (OA), a branch of the internal carotid artery (ICA). The OA enters the orbit and gives rise to ciliary arteries, which supply the choroid and optic nerve head, and the central retinal artery which supplies the retina. The OA and its tributaries, the posterior ciliary arteries and the central retinal artery, provide arterial blood flow to the posterior segment of the globe. The short posterior ciliary arteries from the choroid mainly supply the prelaminar portion of the optic nerve, with a minor contribution to the surface of the disc from fine branches of the central retinal artery. In general, there was a perineural, circular arterial anastomosis (circle of Zinn-Haller) at the scleral level via the short posterior ciliary arteries. Branches from this circle penetrated the optic nerve to supply the prelaminar and laminar regions and the peripapillary choroid [4, 5].

The blood supply to the globe is dependent on these branches of the OA, including the short posterior ciliary and central retinal artery. It is important to realize that the short posterior ciliary arteries in particular supply the choroid and optic nerve head and are vulnerable to changes in systemic blood pressure, perfusion pressure, and vascular dysregulation [6].

## 3. Imaging Analysis of Ocular Blood Flow in NTG

A variety of imaging techniques, as described below, have been used to measure the ocular blood flow. With these technologies, the abnormal ocular blood flow was noticed in NTG patients [7], suggesting that the glaucomatous optic neuropathy might be, at least in part, a result of the impaired autoregulation of ocular vessels.

### 3.1. Fluorescein Angiography

The retinal arteriovenous passage times (AVP) and the perfusion of retinal and choroidal microvascular beds can be qualitatively evaluated by fluorescein angiography. Previous studies found that the AVP was prolonged in NTG patients, indicating that there is a defect in retinal haemodynamics [8, 9]. Furthermore, retinal AVP was correlated with ocular perfusion pressure and systemic blood pressure in NTG patients [8]. Video fluorescein angiograms showed choroidal filling times were also prolonged in NTG patients [10].

### 3.2. Color Doppler Imaging

Color Doppler imaging (CDI) can be used to evaluate erythrocyte velocity of the OA, central retinal artery, and posterior ciliary arteries. CDI provides the end diastolic velocity, the peak systolic velocity, and the mean velocity. A number of studies with CDI demonstrated that the blood flow resistance was increased and blood flow velocities were decreased in the OA and PCAs of patients with HTG, pseudoexfoliation syndrome, and NTG [11, 12], suggesting an important role of ocular blood flow in pathogenesis of glaucomatous optic neuropathy. Reduced blood flow velocities and increased resistive indices in most retrobulbar vessels were found in patients with NTG, which may explain the reason for the optic nerve head fluorescein filling defects and capillary loss of the optic nerve head of these patients [1, 2, 13–15].

### 3.3. Magnetic Resonance Imaging (MRI)

MRI was used to detect the brain ischemic changes in NTG patients, including cerebral white matter lesions and small vessel ischemic damage [16, 17], corpus callosum atrophy in conjunction with cerebral infarcts [18], indicating that the ischemia contributed to glaucomatous neuropathy progression. Deeper depression in the inferior pericentral visual field was found in NTG patients with characteristic ischemic changes on brain MRIs, when compared with those NTG patients of nonischemic change on brain MRIs [19].

### 3.4. Laser Speckle Flowgraphy

As a new noninvasive technology based on the laser speckle phenomenon, laser speckle flowgraphy (LSFG) offers the assessment of microcirculation of the optic nerve head (ONH) and choroid and retinal vessels at the fovea simultaneously [20]. The parameters used in ONH blood flow analysis by LSFG include the waveform variables (like the skew, the acceleration time index, and the blowout time) [21]. LSFG measurement of the ONH revealed the decreased skew and the increased acceleration time index in eyes of patients with mild NTG, when compared with that of normal control and advanced NTG patients. These results support the hypotheses that endothelial dysfunction and/or increased vascular resistance play an important role in pathogenesis of NTG [21].

## 4. NTG and Systemic Disorders

Systemic vascular diseases, including migraine, systemic low blood pressure, Alzheimer's disease, primary vascular dysregulation (PVD), and Flammer syndrome, are associated with the progression of NTG. It is generally accepted that the risk factors of NTG include gender [22, 23], race (more frequently seen in Japan than in European or American countries) [24], PVD [25], and low blood pressure [26, 27]. It is noteworthy that female appeared to be more susceptive to vasospasm and progression of visual field loss in NTG [28]. Similarly, vasospasm was more commonly observed in Japanese than in European and American patients, and, correspondingly, NTG was more commonly seen in Japan than most other countries [29, 30]. Migraine, a disorder associated with NTG, was characterized as a vasospastic disorder and commonly seen in women [31].

### 4.1. Migraine

A number of investigations reported that the migraine was associated with NTG [32, 33]. In the Collaborative Normal Tension Glaucoma Study, migraine was a risk factor for development and progression of NTG [28]. Furlanetto et al. demonstrated that migraine was a predictor for the occurrence of disc hemorrhage of NTG patients in Low-Pressure Glaucoma Treatment Study [33]. In addition, Corbett and his colleagues examined the NTG patients using neurobehavioral testing, neurological history, computerized tomographic scan, and electroencephalography and found that migraine-related ischemia might be the pathogenic mechanism in some cases of NTG, and 44% of these NTG patients had a history of migraine [32]. In another study, migraine was significantly more commonly seen in patients with NTG than control subjects and patients with HTG [31]. These results suggested a potential, common vascular etiology of both NTG and migraine.

Migraine was associated with transient cerebral vasospastic episodes, which in turn resulted in impairment in the mechanisms of autoregulation of blood flow in the central nervous system [34, 35]. Furthermore, NTG patients with silent cerebral infarct had faster rates of visual field deterioration than those with NTG only, suggesting a role of cerebral ischemic injury in glaucoma progression [35]. Autoregulation might be inefficient in patients with glaucomatous optic neuropathy and result in varying degrees of ischemia at the optic nerve [36]. This could predispose one to microinfarction at the optic nerve head and disc hemorrhage [37, 38]. Migraine seems to be a clinical marker of an impaired microvascular autoregulation [33].

### 4.2. Systemic Hypotension

Both systemic hypotension and vasospasm were known as risk factors for glaucomatous damage. A relationship between the incidence of vasospastic disorders and hypotension has been reported [39]. Furlanetto found that low systolic blood pressure was a risk factor for the pathogenesis of disc hemorrhage in NTG [33]. Kaiser et al. showed that the patients with NTG with rapid progression of VF damage and excavation of the optic nerve head had the low systemic blood pressure as a risk factor in common, with 65% of them suffering from vasospasm [27, 39]. Furthermore, a sustained blood pressure drop during sleep was always observed in these patients [27]. It was reported that hypotension, especially nocturnal arterial hypotension, might contribute to progression of NTG because of the optic nerve hypoperfusion [40]. However, Kim et al. showed that systemic blood pressure played a role in the onset of disc hemorrhage in NTG, and the only significant risk factor for disc hemorrhage occurrence in NTG was systemic hypertension [27]. Therefore, systemic hypotension might be a significant risk factor for glaucomatous optic neuropathy.

### 4.3. Alzheimer's Disease

Alzheimer's disease (AD) is a common type of dementia, which is characterized by progressive memory deterioration, cognitive dysfunction, abnormal behavior, and other disorders resulting from central nervous system degeneration. Retinal vessel abnormalities were detected in NTG and early stage of AD [41]. Retinal vessel signs may reflect the vascular dysregulation in retinal and cerebral microvasculature, leading to low perfusion pressure in patients with glaucoma and AD [42, 43]. Sugiyama et al. presented that some of AD patients and NTG patients might share with a common pathologic mechanism [44]. By using single photon emission computed tomography, they compared regional cerebral blood flow (rCBF) in NTG patients with normal individuals and found that 22.6% of NTG patients exhibited an AD-like perfusion pattern although none of them was clinically diagnosed as AD. This incidence rate seemed significantly higher than that rate (1%) of AD reported in a normal population cohort aged 75 and over [44]. Furthermore, the rCBF in the regions of middle cerebral artery perfusion decreased in early stages of AD; meanwhile the levels of rCBF of these NTG patients were lower in these regions than those of controls [44]. Taking into consideration the aforementioned data, a common pathologic mechanism between NTG and AD seems possible.

It was hypothesized that retinal vessel diameters and* Helicobacter pylori* (Hp) and excessive Valsalva might be common risk factors in NTG and AD [41, 45, 46]. Hp, known to be involved in the pathophysiology of glaucoma and AD, may influence the pathophysiology of glaucoma and AD by promoting platelet and leucocyte aggregation [46]. Moreover, Hp eradication appeared to slow down the progression of AD and glaucoma including NTG [47].

### 4.4. Primary Vascular Dysregulation (PVD)

A main cause for the disturbed autoregulation of ocular blood flow was the PVD syndrome that was frequently observed in NTG patients [23]. Female was more likely to be disposed to PVD [48], and, interestingly, patients with PVD were more susceptive to migraines [49, 50], which may explain, at least in part, why female is more susceptive to migraines.

The retinal vessels of PVD subjects usually showed a higher spatial irregularity and higher stiffness [51, 52]. PVD was known to be a main cause for splinter hemorrhages at the border of the ONH, which may explain why in NTG patients ONH hemorrhages often occurred [53, 54]. In terms of circulation, PVD patients had an inborn tendency to respond differently to various stimuli such as cold [29]. Vasoconstriction was the most apparent pathological reaction [23]. In addition, ocular blood flow was correlated with peripheral circulation in PVD patients, as an example, in their fingers [55]. A repeated, but very mild ocular blood flow decrease, mainly due to disturbed autoregulation and ocular perfusion pressure fluctuation, would lead to an unstable oxygen supply and an increased local mitochondrial oxidative stress [56–59]. This process was a recognized pathophysiological mechanism of glaucomatous optic neuropathy.

### 4.5. Flammer Syndrome

Flammer syndrome describes a phenotype characterized by the presence of primary vascular dysregulation together with a cluster of symptoms and signs that may occur in healthy individuals as well as the patients with the diseases [25]. Most subjects with Flammer syndrome were healthy people, which typically showed a number of ocular signs [60]. The retinal vessels were found to be stiffer, with larger spatial variability [60]. The vessel autoregulatory responses to blood pressure and IOP were less sensitive or even absent [25].

Although Flammer syndrome is quite prevalent and mostly benign, it may contribute to the occurrence and progression of potentially serious diseases such as NTG [25]. Generally, patients with progressing glaucomatous damage despite a normal or well-controlled IOP often suffered from Flammer syndrome [25]. Flammer syndrome with NTG hold more signs, including optic disc splinter hemorrhages and diffuse visual field defect, the increased retinal venous pressure and blood flow resistance in retroocular vessels, and increased oxidative stress and activation of retinal astrocytes [25, 60]. In addition, the retinal vessels of the optic nerve head were observed to be less shifted to the nasal side in a number of patients with either Flammer syndrome or NTG [61].

## 5. The Mechanism of Abnormal Ocular Blood Flow in NTG

The mechanisms underlying the abnormal ocular blood flow in NTG remains unclear, but the risk factors for glaucomatous optic neuropathy likely included oxidative stress, vasospasm, and endothelial dysfunction.

### 5.1. The Instability of Blood Flow and Oxidative Stress

Accumulated evidences showed the blood flow instability in NTG patients. The unstable blood flow and unstable oxygen supply resulted in recurrent mild reperfusion injury, which might cause a chronic oxidative stress [62, 63], and particularly affected the mitochondria function of the optic nerve head [23].

Oxidative stress is known to cause an increase in endothelin-1 (ET-1). Various studies demonstrated an increased level of ET-1 in glaucoma patients, particularly in those with progressive neuropathy even though the IOP was well controlled [64, 65]. Metalloproteinases (MMP-2 and MMP-9) were upregulated in the ONH of glaucoma patients. An upregulated MMP-9 was found in circulating lymphocytes [66].

There was a high density of mitochondria in the ONH due to high energy demands [23]. The mitochondria damage causes less efficient energy supply to the ONH. The mild reperfusion will lead to chronic oxidative stress, which in turn specifically affects the structure and function of mitochondria. Other cellular components were affected by oxidative stress. As an example, activated astrocytes responded sensitively to changes in the microenvironment [23].

### 5.2. Vasospasm

Vasospasm may play a key role in ONH damage and lead to systemic autoregulatory dysfunction in NTG patients. Previous studies showed that vasospasm created an environment dysregulation of blood flow, which increased the vulnerability of the ONH to vascular challenges, and this caused perfusion instability, ischemic changes, reperfusion injury, and axonal loss of the ONH [67, 68]. Vasospasm was common and associated with multiple diseases. However, it appeared to be a transient phenomenon that could be reversible by improving retrobulbar hemodynamics in NTG patients using calcium channel blockers [23].

### 5.3. Endothelial Dysfunction

Previous studies showed that the vascular endothelium regulated the microcirculation through release of vasoactive factors, including the vasodilator nitric oxide (NO) and the vasoconstrictor endothelin-1 (ET-1) [69]. NO released from endothelial cells directly stimulated the surrounding vascular smooth muscle to promote vasodilation [70]. Systemic factors such as hyperlipidemia, atherosclerosis, and hyperglycemia impaired endothelial NO signaling through oxidative stress damage [71]. It was known that NO activity contributed to ocular autoregulation and could protect the endothelium and nerve fiber layer against pathologic stresses implicated in glaucoma [70]. Opposing the vasodilation properties of NO was ET-1, the most important and potent vascular constricting factor [72]. A number of studies demonstrated the increased plasma ET-1 levels in NTG patients [73, 74]. In vitro and animal studies showed that ET-1 exerted its vasoconstrictor effects mainly on the microvessels in the retina [75], which could reduce the blood supply to the optic nerve [76]. Buckley et al. identified that dysfunction of systemic vascular endothelial cell caused decreased responsiveness to ET-1 stimulation in NTG patients [67, 77]. Endothelial dysfunction is likely related to NTG, and the endothelial dysfunction may be primary or secondary to vascular diseases including vasospasm and atherosclerosis in its contribution to NTG pathology.

## 6. The Effects of Improving OBF in Glaucoma Patients

Although the elevated IOP is definitely an important risk factor for damage to the optic nerve head (ONH), there is evidence suggesting that compromised tissue blood flow in the ONH is also actively involved in glaucomatous optic neuropathy. Therefore, antiglaucoma drugs that have additional effects on ONH blood flow should have important clinical implications and significance. Previous studies evaluating the effects of the nonselective *β*-adrenergic antagonists, including timolol, carteolol, and betaxolol, on human ONH circulation suggested beneficial influence of long-term carteolol or betaxolol therapy on the ONH circulation [78, 79]. Recent studies indicated that the topical FP-receptor agonists, tafluprost or latanoprost, might significantly increase the blood velocity and blood flow in the ipsilateral ONH both in glaucoma patients and normal volunteers, which cannot be attributed to its IOP reducing effect [80–82]. No doubt these ocular hypotensive eye drops may be worthy of further investigation as neuroprotective medication other than their hypotensive effects on glaucoma patients.

## 7. Conclusion

In summary, the vascular factors are actively involved in pathogenesis of NTG and its related diseases, and OBF plays an important role in the progression of NTG optic neuropathy. The mechanisms underlying the abnormal ocular blood flow in NTG remain unclear, but, likely, oxidative stress, vasospasm, and endothelial dysfunction appear to be the risk factors for glaucomatous optic neuropathy.

Therefore, lowering IOP only for the treatment of glaucoma is not enough; the therapeutic strategy should also include the optic neuroprotection, in which improving OBF should be critical.
